# Encapsulation of drug into mesoporous silica by solvent evaporation: A comparative study of drug characterization in mesoporous silica with various molecular weights

**DOI:** 10.1016/j.heliyon.2021.e08627

**Published:** 2021-12-18

**Authors:** Arif Budiman, Diah Lia Aulifa

**Affiliations:** a Department of Pharmaceutics and Pharmaceutical Technology, Faculty of Pharmacy, Universitas Padjadjaran, Jl. Raya Bandung-Sumedang Km. 21, Indonesia; b Department of Pharmaceutical Analysis and Medicinal Chemistry, Faculty of Pharmacy, Universitas Padjadjaran, Jl. Raya Bandung-Sumedang Km. 21, Indonesia

**Keywords:** Mesoporous silica, Molecular weights, Itraconazole, Nifedipine, Nicotinamide

## Abstract

Mesoporous silica (MS) is a promising material as a drug carrier that is used in pharmaceutical applications. It was discovered that the incorporation of drugs into MS has the potential to improve their dissolution and bioavailability due to the large specific surface area. This study aimed to characterize the drugs with various molecular weights in MS as well as to elucidate their impact on the loading amount and the amorphization within MS. The solvent evaporation method was used to encapsulate itraconazole (ITZ), nifedipine (NIF), and nicotinamide (NIC), respectively, into MS. The result shows the absence of glass transition and the melting peak of ITZ, NIF, and SAC within MS signifying the successful encapsulation. A hallo pattern was found in ITZ and NIF within MS indicating the amorphization. The high molecular weight and the interaction between the drug with the silica surface is reportedly contributed to the formation of the amorphous state. Meanwhile, the characteristic diffraction peaks of NIC crystal were observed for NIC within MS. In conclusion, the molecular weight of the drug has a significant effect on the loading amount and the amorphization of the drug within MS.

## Graphical abstract

**Figure:**
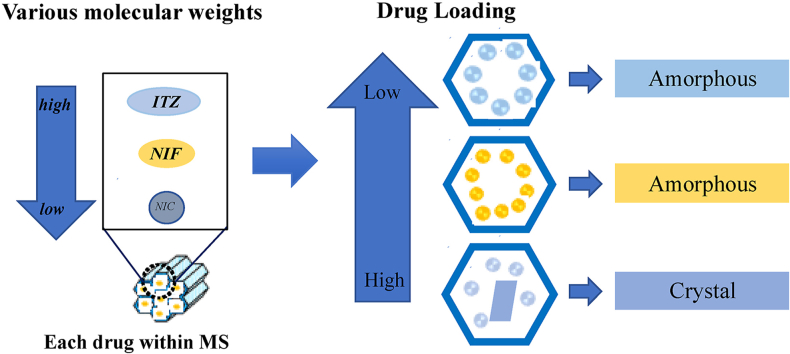

## 1 Introduction

The aqueous solubility of active pharmaceutical ingredient (API) has a strong influence on its bioavailability when orally administrated (Budiman et al., 2021). Recently, about 90% of drug candidates, currently under development, possess low aqueous solubility and high hydrophobicity characteristics (Dening and Taylor, 2018). Therefore, formulation strategies are required to enhance drug solubility and improve absorption from the gastrointestinal tract, especially in the formulation of oral administration (Skorupska et al., 2015). Some of the strategies adopted to improve oral bioavailability include the use of additives such as surfactants or complexing agents, salts (Takano et al., 2010), and amorphous solid dispersions (Almeida E Sousa et al., 2016).

As an alternative, mesoporous silica (MS) is a popular strategy in drug delivery systems due to its ability to enhance dissolution and their absorption from the gastrointestinal tract (Dening et al., 2019; Mellaerts et al., 2007). Furthermore, the encapsulation of drugs into MS is carried out to stabilize and deliver drugs in an amorphous state via the oral route (Xu et al., 2013). MS has large specific surface areas that lead to a high surface free energy, therefore, the absorption of the drug on the surface of MS allows the system to progress to a lower free energy state (Mulikova et al., 2021; Qian et al., 2015; Rengarajan et al., 2008). The physical stability of the amorphous drugs in MS was attributed to a decrease in the Gibbs free energy of the system due to adsorption and the size-constraint effect of MS on drug crystal growth (Dening and Taylor, 2018).

The chosen method for encapsulation into MS has a significant effect on the efficiency of drug loading. Furthermore, drugs must be temporarily mobilized by either the solvent-free (melt method) or solvent-based methods (incipient wetness impregnation, solvent evaporation method) to ensure encapsulation (Le et al., 2019; Riikonen et al., 2018). Moreover, several factors influence drug loading including pore size and volume, surface area and surface functionality of MS, loading method, surface interaction, and drug molecule size (Ambrogi et al., 2007; Le et al., 2019; Vraníková et al., 2020).

Many studies have reported the encapsulation of drug into mesoporous silica. However, a comparative study of drug characterization with various molecular weights encapsulated into mesoporous silica have not been studied, moreover their impact on the loading amount and the amorphization of drug within mesoporous silica is not clearly understood. Therefore, this study aimed to characterize drugs with various molecular weights encapsulated into MS, and elucidate its impact on the loading amount and the amorphization of drug within MS. The solvent evaporation method was adopted for the encapsulation of the drug into MS using chloroform as the solvent. This method is an attractive approach due to its high efficiency and familiar unit operations such as filtration and drying. Meanwhile, chloroform was chosen as the solvent due to its efficiency in drug loading (Šoltys et al., 2019).

## 2 Materials and methods

### 2.1 Materials

**Figure 1:**
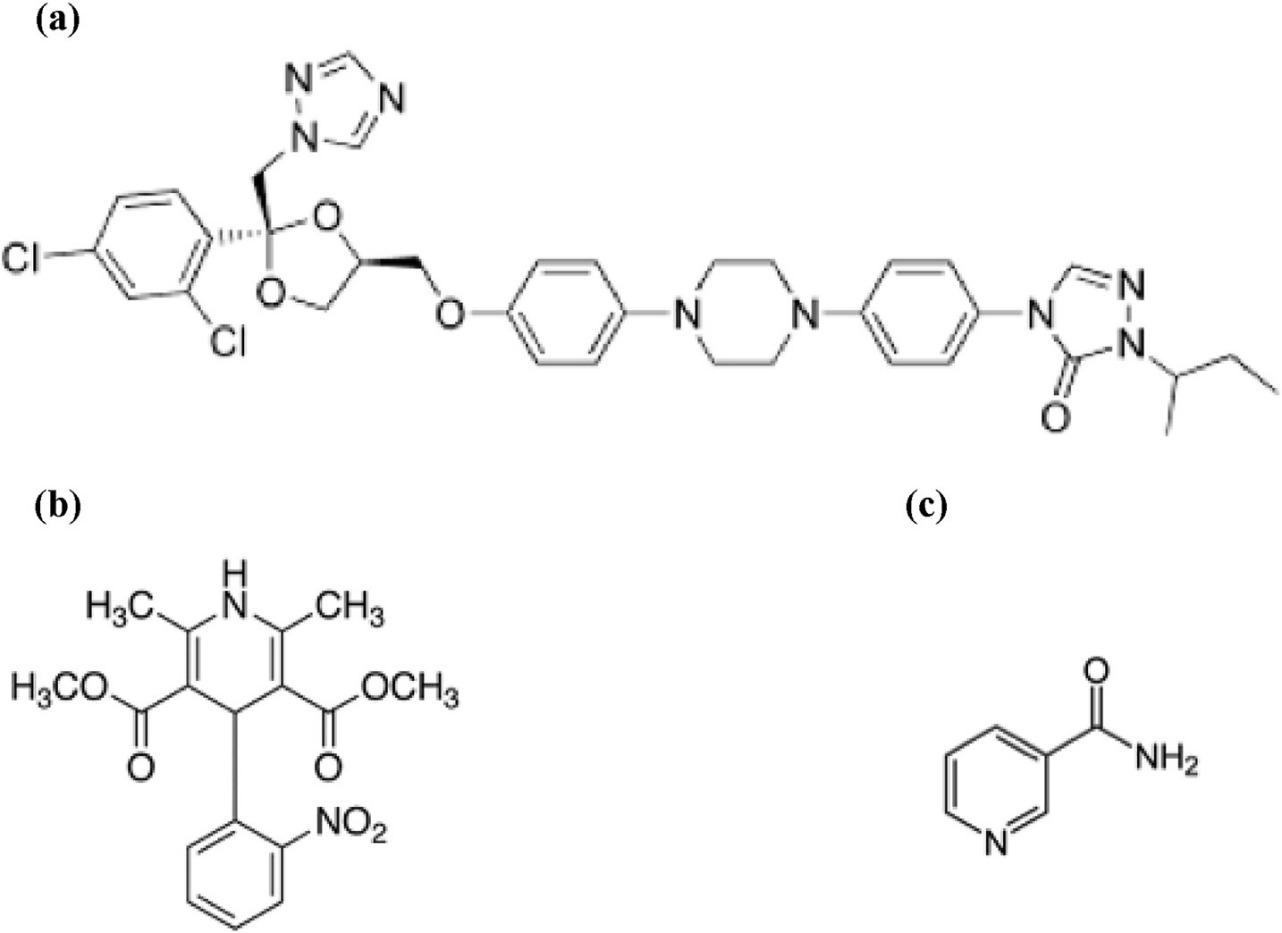
Chemical structures of (a) itraconazole (ITZ), (b) nifedipine (NIF), and (c) nicotinamide (NIC).

ITZ (MW = 705.64 g/mol), NIF (MW = 346.33 g/mol) and NIC (MW = 122.18 g/mol) were purchased from FUJIFILM Wako Pure Chemical Corporation (Osaka, Japan). Their chemical structures are represented in Figure 1 and MS was kindly gifted from Taiyo Kagaku., Ltd. (Mie, Japan).

### 2.2 Methods

#### 2.2.1 Morphology and structure characterization

The morphology and the porous structure of MS samples were obtained by a TEM (JEM-1400, JEOL Ltd., Japan). Prior to analysis, samples were dispersed into ethanol, sonicated for 10 min, and then adsorbed on carbon-plated copper grids.

#### 2.2.2 Preparation of each drug encapsulated into MS

Each drug was dissolved in chloroform and then, MS was dispersed in a chloroform solution containing the drug with various weight ratios. The suspension was sonicated at 25 °C for 3 min and evaporated for 30 min at 30 °C using a rotary evaporator with a water bath. The resulting powder was dried at 30 °C for 48h using a vacuum dryer to obtain ITZ encapsulated into MS (ITZ/MS), NIF encapsulated into MS (NIF/MS), and NIC encapsulated into MS (NIC/MS).

#### 2.2.3 Modulated differential scanning calorimetry (MDSC) measurement

MDSC measurement was carried out using a DSC-7000X instrument and approximately 5 mg of the sample was placed into a crimped aluminum DSC pan under a N_2_ purge at a flow rate of 50 mL/min. Afterward, the samples were measured from 0 to 200 °C at a heating rate of 2 °C/min with modulation of ±0.5 °C every 60s.

#### 2.2.4 Powder X-ray diffraction PXRD measurement

The PXRD patterns were collected using a Miniflex II with the following conditions: target, Cu; filter, Ni; voltage, 30kV; current, 15mA; scanning rate, 4°/min, and scanning angle of 2θ = 3°–40°.

#### 2.2.5 Fourier transform-infrared (FT-IR) spectroscopy

FT-IR measurements were carried out on FT-IR spectrometer (Bruker Optik GmbH, Ettlingen, Germany), using KBr tablet method. FT-IR spectra were obtained in the scan range of 400–4000 cm^−1^ at a resolution of 4 cm^−1^.

## 3 Result and discussion

### 3.1 Morphology of MS

**Figure 2:**
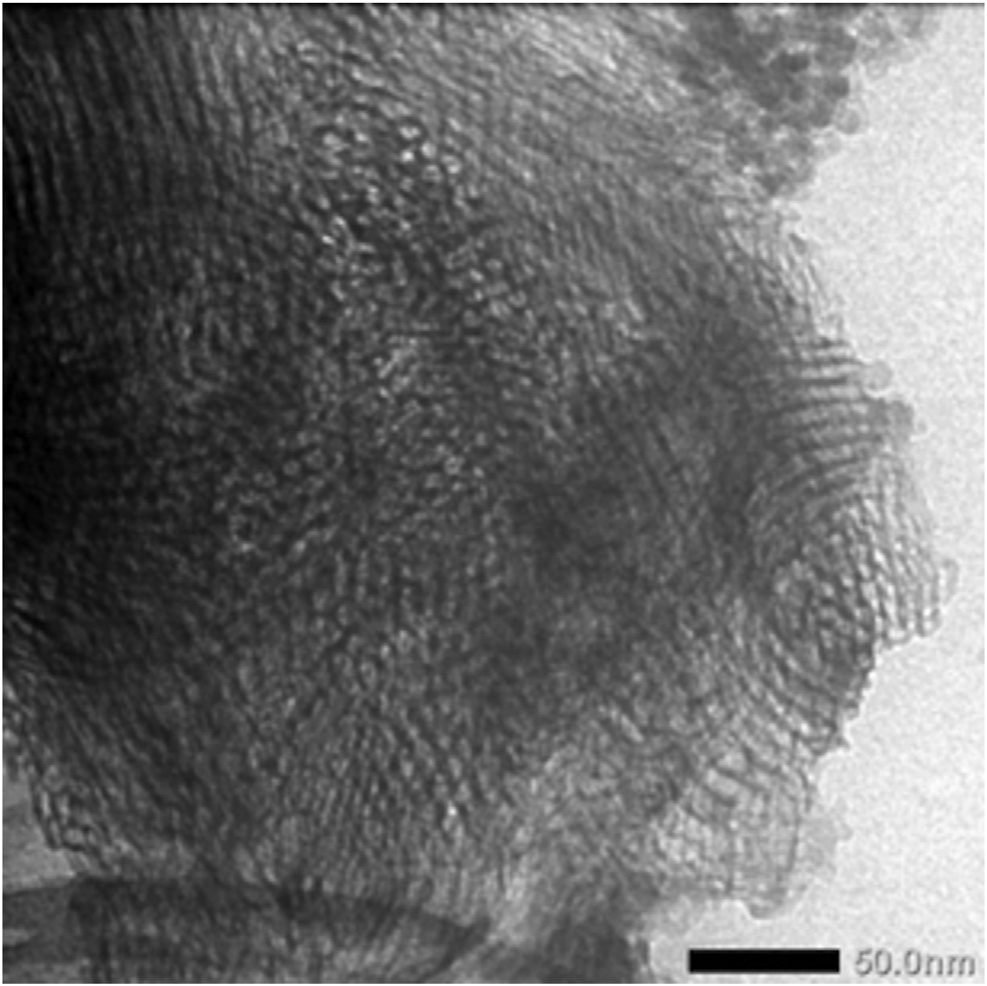
TEM image of MS nanoparticles.

Morphology of MS was studied by TEM to observe the well mesostructure. Figure 2 showed ordered mesoporous silica and a porous texture in accordance with the mesoporous silica materials. MS presented a typically irreversible type IV isotherm, according to the IUPAC classification. The specific surface area, pore-volume, and pore diameter of MS were 820 nm^2^/g, 0.92 cm^3^/g, and 8nm, respectively.

### 3.2 Encapsulation of ITZ into MS

**Figure 3:**
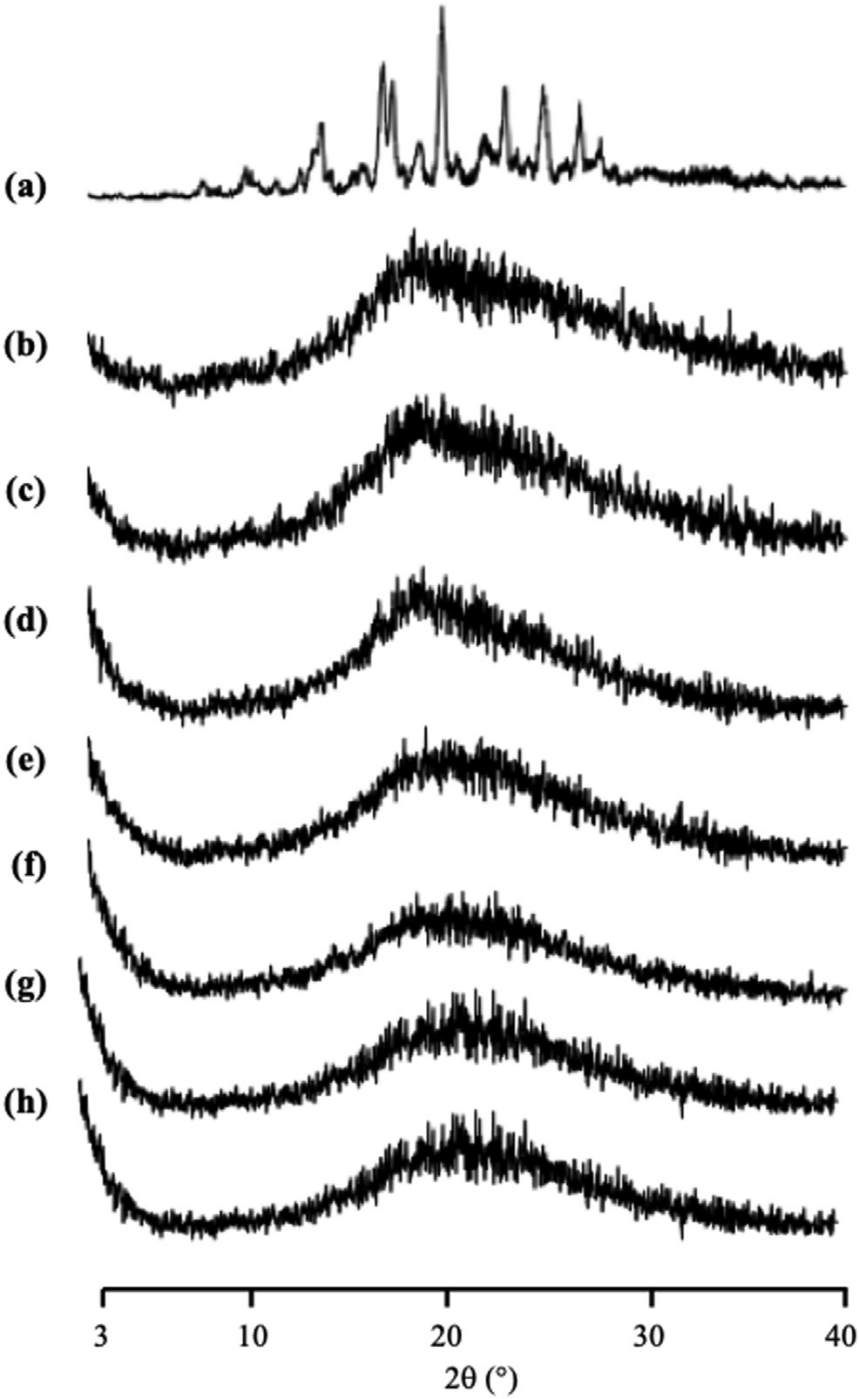
The PXRD patterns of (a) ITZ crystal, ITZ/MS = (b) 9:1, (c) 8:2, (d) 7:3, (e) 6:4, (f) 5:5, (g) 4:6, and (h) 3:7.

The ITZ/MS was evaluated by PXRD as shown in Figure 3 and its crystals showed characteristic diffraction peaks in the PXRD patterns. In contrast, the ITZ/MS at various weight ratios showed almost a hallo pattern without any diffraction peak. This indicated that the ITZ crystal was amorphized by solvent evaporation. Furthermore, the characteristic diffraction peaks of ITZ crystal in the ITZ/MS with the higher weight ratios were not observed although some ITZ was outside of MS. This is due to the crystallization tendencies of ITZ categorized into class III base on Taylor's classification, which does not crystallize easily during storage (Baird et al., 2010; Budiman et al., 2021). Base on this result, the PXRD measurement is unable to determine the maximum amount of ITZ encapsulated into MS.

**Figure 4:**
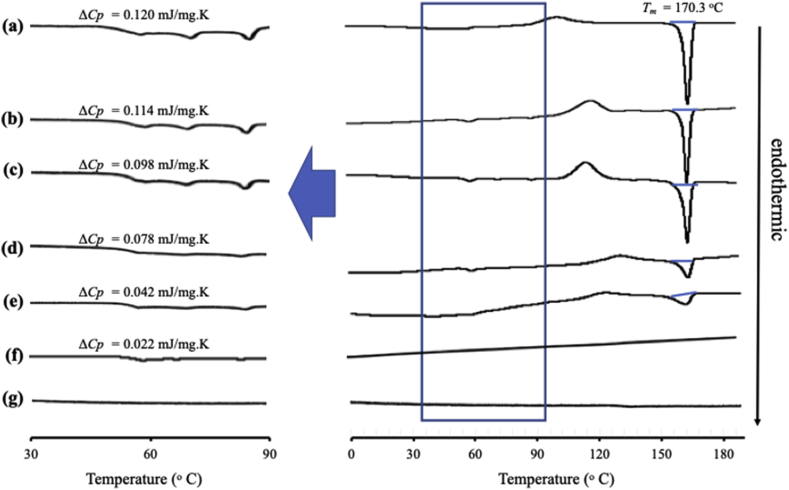
(left) Expanded and (right) full MDSC curve of ITZ/MS = (a) 9:1 (b) 8:2 (c) 7:3 (d) 6:4 (e) 5:5 (f) 4:6 and (g) 3:7.

MDSC measurements were conducted to determine the loading amount of drug into MS (Figure 4). The DSC curve of crystalline ITZ exhibited an endothermic peak at 170.3 °C, which corresponds to its melting point, while the glass transition temperature (*T*_*g*_) of ITZ amorphous was 56.7 °C. There was no melting peak and *T*_*g*_ in MDSC curves of MS between 0 and 190 °C. Two ITZ endothermic liquid crystal of was observed with initial temperature values for a smectic to nematic transition at 72.1 °C and a nematic to isotropic transformation at 88.2 C (Kozyra et al., 2018). The melting peak of ITZ crystal was observed in ITZ/MS with weight ratios of 9:1–5:5 and ITZ heat of fusion dwindle with a decrease in ITZ concentration, however, the melting peak disappeared in ITZ/MS at a ratio of 3:7. The drugs encapsulated into MS show no melting peaks in DSC curves due to their amorphous form (Tanabe et al., 2012). Therefore, the absence of a melting peak of ITZ in ITZ/MS at a ratio of 3:7 indicated that ITZ was encapsulated into MS (Liu et al., 2016). A previous study reported that the absence of *T*_*g*_ was due to the monomolecular adsorption of the drug on the silica surface. A similar result in melting peak was observed with a glass transition of ITZ in ITZ/MS with weight ratios of 9:1–4:6, while in the weight ratios of 3:7 the *T*_*g*_ was not detected. The presence of *T*_*g*_ for ITZ/MS indicated that some of ITZ amorphous were unloaded in MS (Budiman et al., 2021).

**Figure 5:**
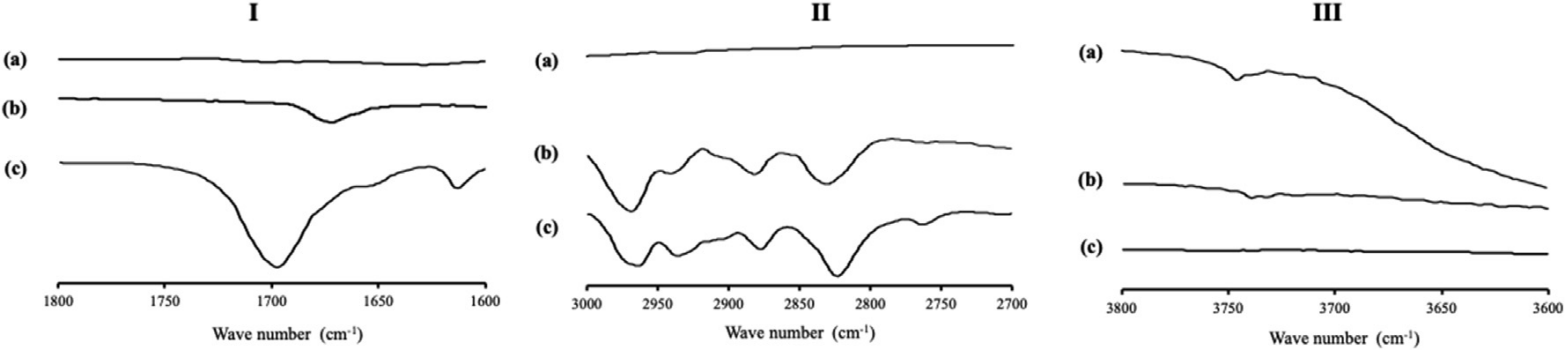
FT-IR spectrum of (a) MS, (b) ITZ/MS, and (c) ITZ, in the carbonyl stretch region (I), the CH stretch region (II), and the OH stretch region (III).

The carbonyl of ITZ present electronegative oxygen centers, thus the interaction of ITZ and MS can be predicted through hydrogen bonding between the electrons lone pairs associated with nitrogen atoms and surface silanol groups. The FT-IR spectroscopy was conducted to confirm the interaction between ITZ and MS (Figure 5). After being incorporated into MS, a free carbonyl vibration around 1700 cm^−1^ shifted to around 1675 cm^−1^ indicating the possibility of the hydrogen bonding between the carbonyl oxygen atom with the surface hydroxyl. MS exhibited a characteristic signal at 3750 cm^−1^ attributed to the stretching vibrations of isolated (i.e. non-hydrogen bonded) silanol groups. The incorporation of ITZ into MS caused a bathochromic shift of the silanol vibrations due to hydrogen bonding with ITZ carbonyl-groups, although the peak intensity has decreased. The bathochromic shift was also observed in the CH stretching region which attributed to the interaction of the neighboring ether and piperazine groups with silanols. These findings demonstrated that the amorphization of ITZ in MS was not only occurred due to the nanoconfinement effect of MS but also due to surface interaction between ITZ and MS.

**Figure 6:**
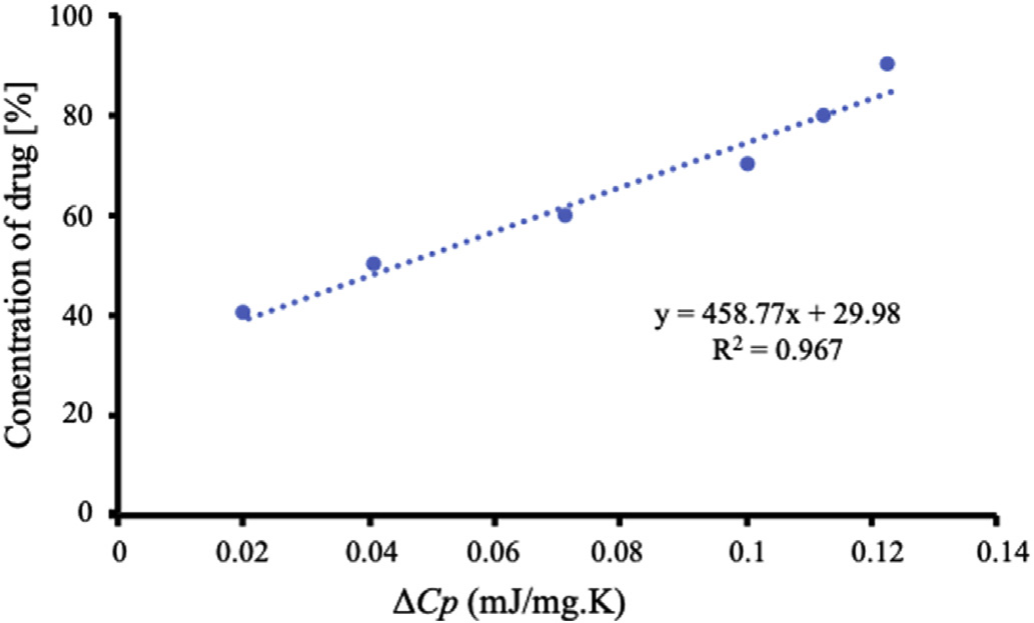
Plots of the concentration of ITZ against Δ*Cp* of ITZ amorphous calculated from the MDSC curves.

The maximum amount of ITZ encapsulated into MS can be estimated quantitatively by Δ*Cp* ITZ amorphous on the DSC curves. The concentration of ITZ was plotted as a function of the Δ*Cp* as shown in Figure 6. The fitted lines for ITZ/MS systems show good linearity with correlation coefficients of 0.97. The y-intercept value represents the maximum amount of ITZ that is encapsulated into MS which was 29.98 %. This result was almost similar with the calculation of theoretical monolayer coverage of ITZ within MS, in which the amount of ITZ required for monomolecular adsorption of MS surface was 29.93 w/w%.

### 3.3 Encapsulation of NIF into MS

**Figure 7:**
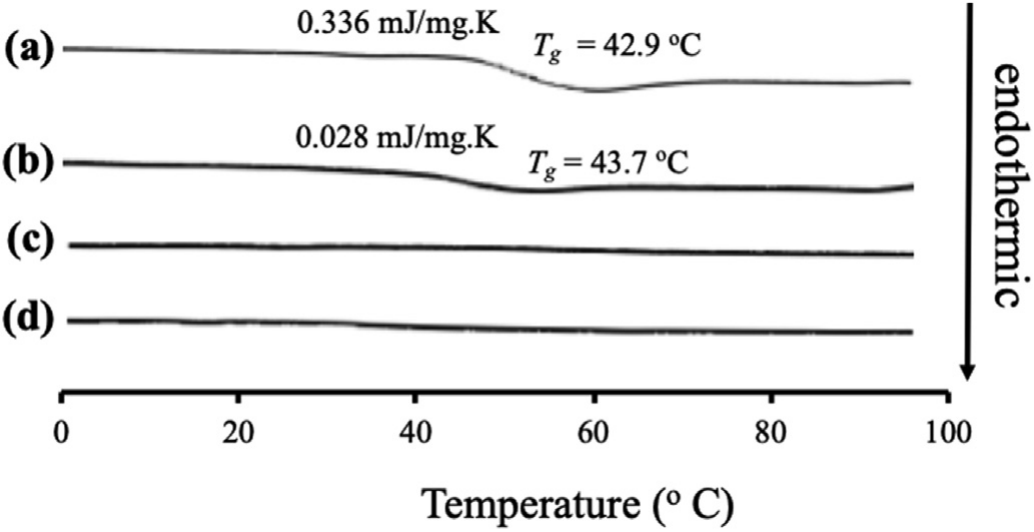
MDSC curve of (a) NIF amorphous, NIF/MS = (b) 5:5, (c) 4:6, and (d) 3:7.

The NIF/MS at various weight ratios showed a similar result with ITZ/MS with an observed hallo pattern without any diffraction peak. This indicated that the NIF crystal was amorphized by solvent evaporation (data not shown) and the MDSC curves of the NIF/MS system are shown in Figure 7. The crystalline NIF showed an endothermic peak at 171.3 °C, while the *T*_*g*_ of NIF amorphous was 42.9 °C. In the weight ratio of 5:5, the *T*_*g*_ of NIF was observed indicating that some amorphous NIF were unloaded in MS (Budiman et al., 2021). In contrast, the *T*_*g*_ was not detected in the weight ratio of 4:6 indicating an encapsulation of NIF into MS. In a previous study, it was reported that TV of NIF was observed even though the NIF was incorporated into controlled pore glasses (CPG) with a size of 7.5, which is similar to the size of the pores of MS, although the *T*_*g*_ decrease. Furthermore, the *T*_*g*_ of NIF was reduced by nanoconfinement due to an intrinsic size effect indicating that the mobility of NIF in MS was higher than amorphous NIF (Cheng and McKenna, 2019). However, in this study, the *T*_*g*_ of NIF was not observed after incorporating into MS, even in the weight ratio of 4:6. It is assumed that the absence of *T*_*g*_ was attributed to the interaction between NIF and the silica surface of MS forming the monolayer adsorption of NIF on the silica surface (Mellaerts et al., 2007; Van Speybroeck et al., 2010).

**Figure 8:**
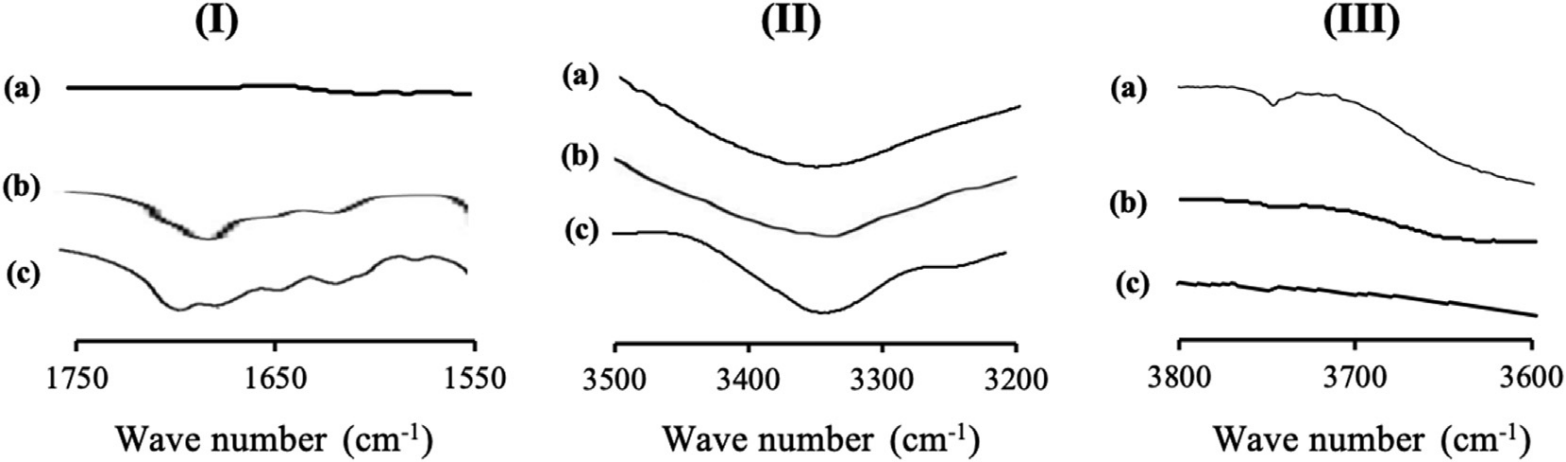
FT-IR spectrum of (a) MS, (b) NIF/MS, and (c) NIF, in the carbonyl stretch region (I), the N–H stretch region (II), and the OH stretch region (III).

The FT-IR spectra of NIF/MS is shown in Figure 8. NIF amorphous showed C=O stretching peaks at around 1700 cm^−1^ and N–H stretching peak at 3350 cm^−1^. NIF amorphous forms hydrogen bonds with another NIF molecule through one of the C=O groups and the N–H group. The different C=O stretching peaks at 1700 cm^−1^ in the amorphous NIF are attributed to the hydrogen-bonded and non-hydrogen-bonded C=O groups of NIF, respectively. In NIF/MS, the relative peak intensity of the hydrogen-bonded C=O group of NIF was reduced compared with the NIF amorphous. Meanwhile, the N–H stretching peak of NIF was not clearly observed due to the overlap with the peak of MS. This may be due to the spectra intensity of NIF/MS being dominated by silica vibrations, which are spanned the entire wavenumber range. Thus, the characterization of NIF within MS, especially about the interaction of NIF with silica surface was still unclear. However, the characteristic signal at 3750 cm−1 attributed to silanol groups of MS disappeared for NIF/MS. The disappearance of the isolated silanol stretching band in NIF/MS suggested the interaction between NIF molecules with silanol groups of MS.

### 3.4 Encapsulation of NIC into MS

**Figure 9:**
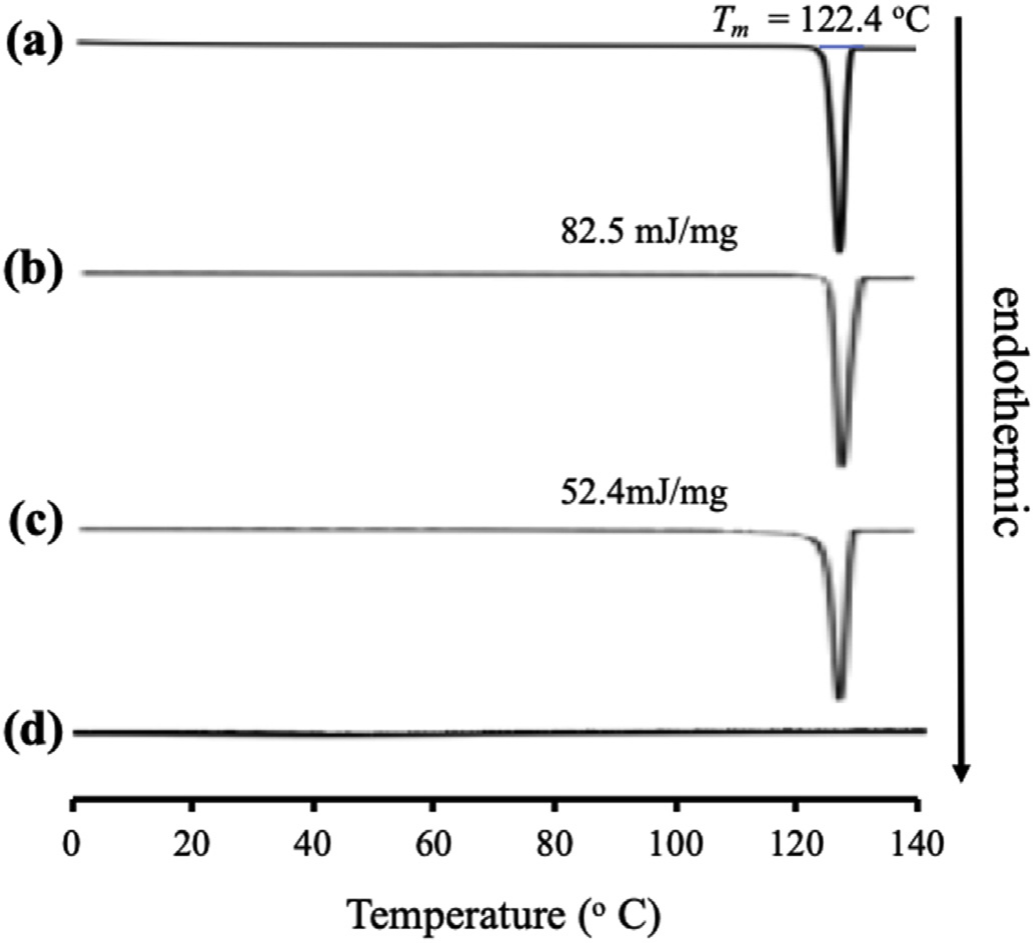
MDSC curve of (a) NIC crystal, (b) evaporated sample of NIC, NIC/MS = (c) 8:2, and (d) 5:5.

The MDSC curve of NIC/MS with various weight ratios is shown in Figure 9. The crystalline NIC showed an endothermic peak at 126.8 °C, while the *T*_*g*_ of NIC was not detected even though it was prepared by the solvent evaporation method. This is due to the high crystallization tendencies of NIC categorized into class I based on Taylor's classification. The melting peak of NIC crystal was observed in NIC/MS at a ratio of 8:2. In contrast, it disappeared in ITZ/MS with a weight ratio of 5:5. The absence of a melting peak of NIC also indicated the encapsulation of NIC into MS (Liu et al., 2016).

**Figure 10:**
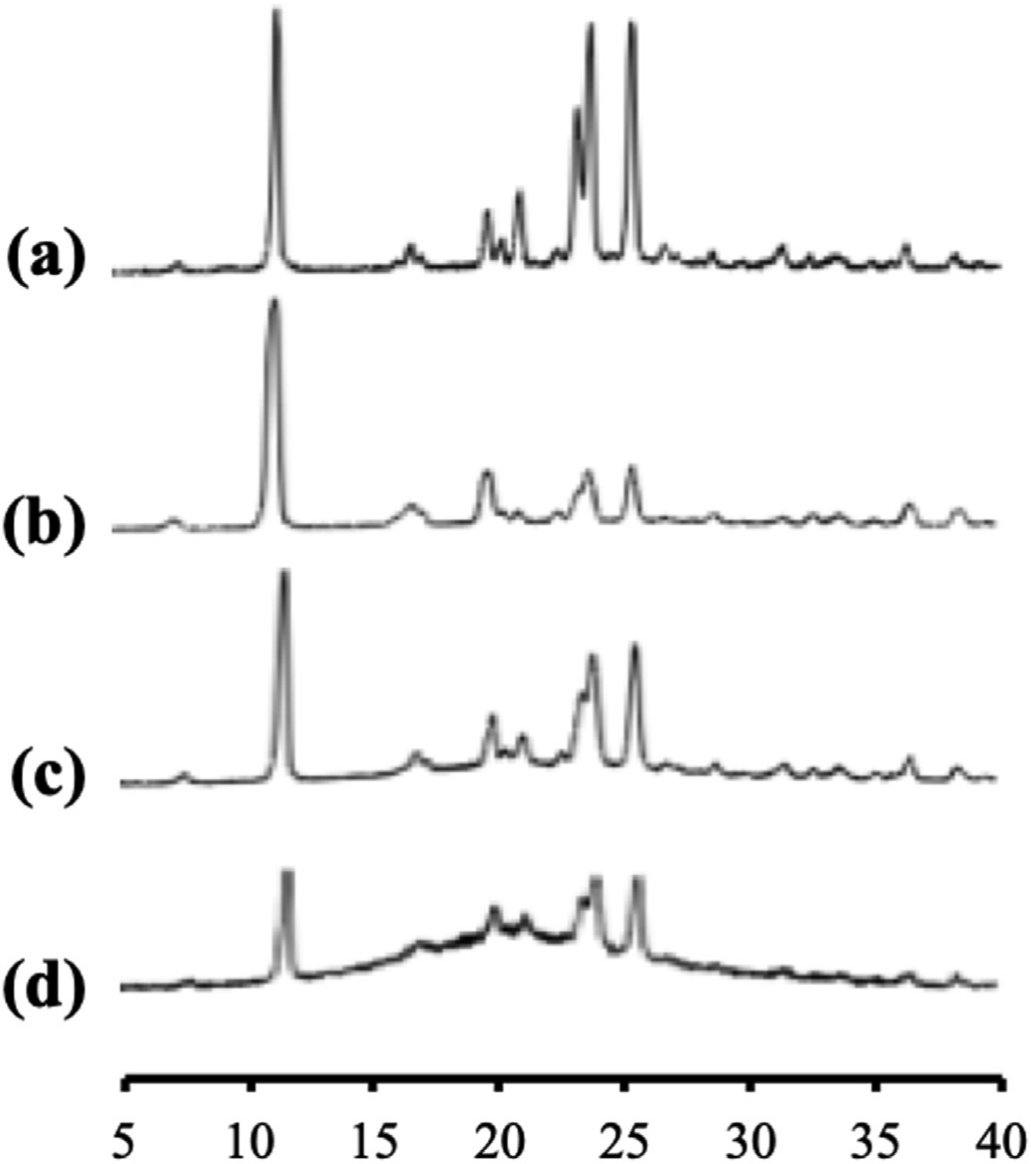
PXRD pattern of (a) NIC crystal, (b) evaporated sample of NIC, NIC/MS = (c) 8:2, and (d) 5:5.

Figure 10 showed the PXRD pattern of NIC encapsulated into MS. The diffraction peaks characteristic of crystalline NIC were observed in both the intact NIC intact and the evaporated NIC samples. Interestingly, the NIC/MS system showed diffraction peaks characteristic in the PXRD patterns even in the weight ratio of 5:5. The crystallization of NIC both within and outside MS. Furthermore, the crystallization of NIC within MS occurred because there was none or weak interaction between NIC and the silica surface, which led to the recrystallization of NIC. Moreover, the difference between the NIC molecule and the pore size is extremely high, therefore, the critical nucleus size of NIC was formed within MS, which led to subsequent recrystallization (Budiman et al., 2021).

**Figure 11:**
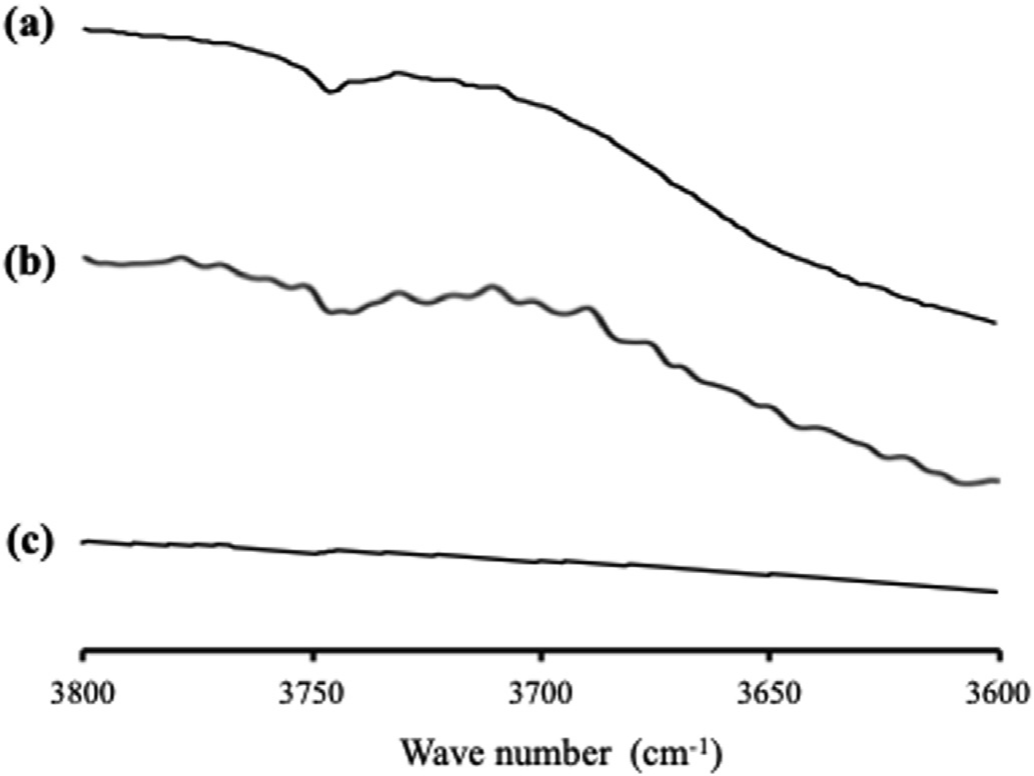
FT-IR spectrum of (a) MS, (b) NIC/MS, and (c) NIC, in the OH stretch region.

The FT-IR measurement was also carried out to confirm the interaction between NIC and MS. The C=O stretching vibration and N–H stretching peaks of NIC is assigned at 1700 cm−1 and 3364 cm−1, respectively. In the NIC/MS, the same spectrum characteristics with NIC was observed, although the peak intensity has significantly decrease (data not shown). Moreover, the characteristic signal at 3750 cm−1 which attributed to the stretching vibrations of silanol groups still remains in NIC/MS (Figure 11). These results indicated that there was none or weak interaction between NIC and the silica surface, which was in agreement with the result of PXRD measurement.

## 4 Discussion

**Figure 12:**
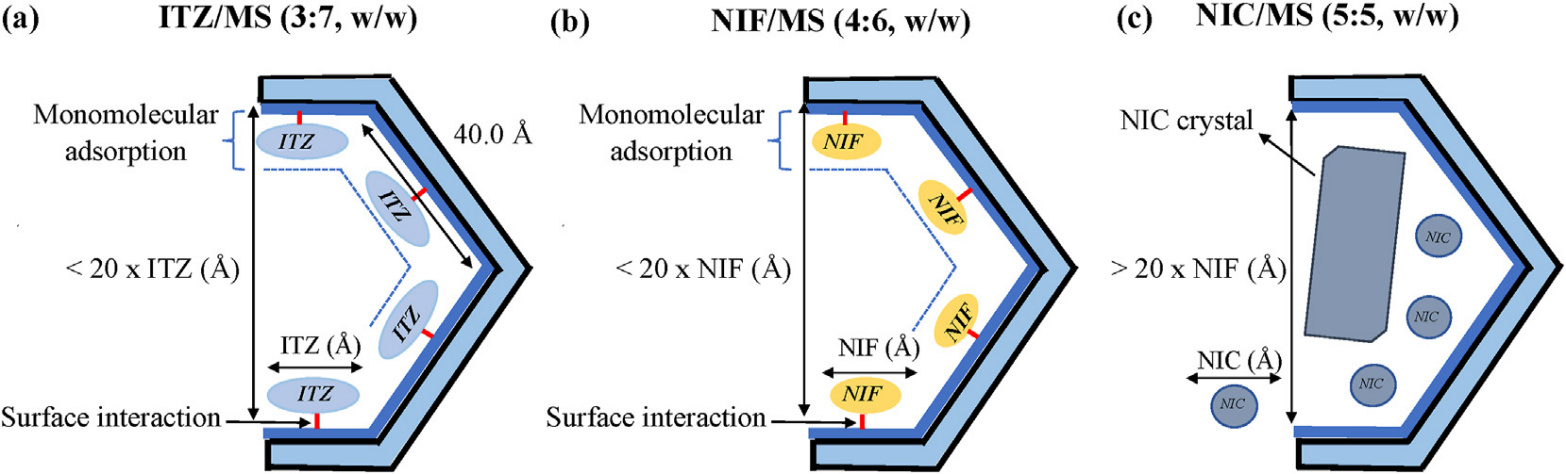
Schematic illustration of (a) ITZ/MS = 3:7, (b) NIF/MS = 4:6, and (c) NIC/MS = 5:5.

A speculated mechanism of each drug within MS based on its characterization evaluated was proposed in the present study (Figure 12). The size of the drug molecule as well as the interaction between drug and silica surface influence the maximum loading amount and the amorphization of drug within MS. A previous study reported that the drug within nanoconfinement has a different *T*_*g*_ compared to the amorphous drugs in the bulk state. The *T*_*g*_ was generally reduced in nanoconfinement due to an intrinsic size effect, indicating higher mobility of drug in a porous structure compared to the bulk state. However, the present study showed that the *T*_*g*_ of drugs were not detected after being encapsulated into MS, particularly for the 3:7 ratio of ITZ/MS and 4:6 of NIF/MS. The monomolecular absorption of ITZ and NIF on the silica surface is due to the absence of *T*_*g*_ in MDSC curves. The theoretical monolayer coverage of ITZ within MS can be calculated using the following Eq. (1).

(1) $$X=\;\frac{\;SSA_{\text{MS}}\;\times \;10^{20\;}\times \;MW_{\text{ITZ}}\;}{S_{\text{ITZ}}\;\times \;N_{\text{A}}}$$

where *X* is the capacity of ITZ required for monolayer coverage of MS (g/g), *MW*_ITZ_ is the molecular weight of ITZ (705.64 g/mol), *S*_ITZ_ is the molecular contact surface area of ITZ (261 Å^2^) (Mellaerts et al., 2011), *SSA*_MS_ is the specific surface area of MS (820 m^2^/g), and $N_{\text{A}}$ is the number of Avogadro. Based on this equation, the theoretical amount of ITZ required for monolayer coverage of MS was 29.93 w/w% (Budiman et al., 2021; Dening and Taylor, 2018). Therefore, the incorporation of 30% ITZ into MS in the experiment was quite reasonable due to the similarity with the theoretical value. Moreover, this result suggested that the carbonyl and ether groups of ITZ interacted through hydrogen bonding with surface silanol groups, which was in agreement with the previous study (Mellaerts et al., 2011). ITZ was also amorphized by encapsulation into MS. The crystallization of ITZ was not observed in MS due to the size difference between the molecule of ITZ and the pore size of MS was not significant. It was reported that the drug recrystallization within the MS was observed when the pore size of MS was 20 times larger than the molecular size of the drugs (Ambrogi et al., 2007). The pore size of MS at 8.0nm is 4–5 times larger than ITZ, which is 2.61 nm (Mellaerts et al., 2011). Therefore, the free space of MS was not sufficient for ITZ to form the critical nucleus size, which is the subsequent crystallization of ITZ (Budiman et al., 2021).

Similar to the ITZ/MS ratio of 3:7, the hydrogen bond between NIF molecules and silanol groups of MS was also suggested. Although NIF was categorized into class II base on Taylor's classification and the crystallization of NIF was not observed in MS. A previous study reported a crystallization of NIF within controlled pore glass at a size above 22.6nm, while no crystallization was detected at 7.5nm and 12 nm, which is similar to the pore size of MS. The nanoconfinement effect of MS inhibits the formation of the critical nucleus size and crystallization of NIF (Ambrogi et al., 2007; Cheng and McKenna, 2019).

In contrast, in the NIC/MS ratio of 5:5, the crystallization of NIC occurred within MS. It was suggested that the pore size of MS was more than 20 times compared to the molecule size of NIC, which led to the crystallization of NIC within MS. Moreover, there is none or weak interaction between NIC and the silica surface that contributed to the crystallization of NIC within MS.

## 5 Conclusion

This study clearly explains the characterization of ITZ, NIF, and NIC encapsulated into MS using the solvent evaporation method. Furthermore, the measurement of PXRD revealed that ITZ and NIF were amorphized within MS, while NIC recrystallize. The maximum amount of each drug encapsulated into MS is then determined by MDSC. Meanwhile, the molecular size and the interaction between the drug with the silica surface contributed to the formation of the drug in the amorphous state. This study provided fundamental insight that the loading amount and the amorphization of drugs within MS depend on the molecular weight of drugs.

## Declarations

### Author contribution statement

Arif Budiman: Conceived and designed the experiments; Performed the experiments; Analyzed and interpreted the data; Contributed reagents, materials, analysis tools or data; Wrote the paper.

Diah Lia Aulifa: Conceived and designed the experiments; Analyzed and interpreted the data.

### Funding statement

This research did not receive any specific grant from funding agencies in the public, commercial, or not-for-profit sectors.

### Data availability statement

Data will be made available on request.

### Declaration of interests statement

The authors declare no conflict of interest.

### Additional information

No additional information is available for this paper.
